# Exploring healthcare personnel’s knowledge, barriers, and innovative approaches in personalised oncology medicine: a scoping review

**DOI:** 10.1080/09581596.2025.2520410

**Published:** 2025-06-28

**Authors:** Shibu Shrestha, Gemma Watts, Susi Geiger

**Affiliations:** ^a^ School of Population Health, RCSI University of Medicine and Health Sciences, Dublin, Ireland; ^b^ School of Business, University College Dublin, Dublin, Ireland

**Keywords:** Attitudes, barriers, healthcare personnel, oncology, personalised medicine

## Abstract

Personalised medicine is widely utilised in oncology, and healthcare personnel are its main gatekeepers and implementers. This scoping review provides insights into the knowledge and attitudes of healthcare personnel toward personalised medicine for cancer, barriers and challenges faced, and innovative practices employed for the provision of personalised medicine. Extensive database searches identified 19,972 studies, of which 50 studies were included in the final review. The data was charted by two reviewers and analysed thematically. The knowledge of healthcare personnel of personalised medicine was mixed, with some studies reporting overall good knowledge (*n* = 2) while some reported poor knowledge among healthcare personnel (*n* = 4). There was high interest (63–95%) in furthering education and training in personalised medicine (*n* = 6). The commonly reported barriers and challenges were: limited reimbursement and insurance coverage mechanism (*n* = 11); insufficient education and training (*n* = 10); and lack of trained personnel to provide the service (*n* = 7). The innovations identified emphasised enhancing the skills and capacity of the existing workforce as well as using technologies to assist in timely decision-making. Overall, gaps were identified at the human resource, institutional, and systemic levels, which will need to be addressed to improve the provision of personalised medicine and healthcare personnel’s confidence levels.

## Introduction

Personalised medicine (PM) refers to tailoring medical treatment based on the individual (mostly genetic) characteristics of patients (Mishra et al., 2019). It is considered to be the next level in patient care, ‘aiming for the right treatment for the right patient at the right time’ (Jackson & Chester, 2015, p. 262). While the exact nomenclature remains fluid, PM is often referred to as precision medicine, individualised medicine, or stratified medicine as well as genomic/genetic medicine/service/testing or pharmacogenetics/pharmacogenomics (Ali-Khan et al., 2016; American Cancer Society, 2022; Ashley, 2015; De Grandis & Halgunset, 2016). PM has been proven to be extremely useful for various diseases including lung cancer, brain tumour, prostate cancer, rheumatoid arthritis, and autoimmune diseases (Gameiro et al., 2018; Mishra et al., 2019).

Healthcare personnel (HCP) – primarily nurses, clinicians, but also broader support team members – are both gatekeepers and implementers of PM therapies. They are responsible for recommending these therapies to their patients; assisting in informed decision making; educating patients; administering therapies and managing care of patients (Spanakis et al., 2020). However, research has indicated that HCPs have limited knowledge and awareness of PM, limiting them from effectively enacting their gatekeeper role (Delikurt et al., 2015; Diamonstein et al., 2018). Previous studies have also reported that HCPs’ attitudes influence the uptake of medical procedures, thereby potentially affecting their implementation into clinical care (Vetsch et al., 2019). Given HCPs’ centrality in the adoption of PM, their lack of confidence and education may be causally related to the relatively low PM adoption rates observed (Farmaki et al., 2024). The shift towards PM will thus require significant advances in the expertise and skills of HCP (Spanakis et al., 2020), requiring redesigning of the medical education curriculum and training (Gameiro et al., 2018). Such knowledge building will also need to take account of global differences in the skills and capacity of HCPs, with research indicating that knowledge gaps may be exacerbated in lower-income countries (LMICs) (Adeniji et al., 2021). Therefore, an assessment of HCPs’ current knowledge base of and experience with PM may be an important indicator as to the future of PM adoption, and identification of existing barriers and innovative practices may give vital directions in improving adoption rates.

The objective of this review is to conduct an analysis of HCPs’ knowledge, confidence and attitudes in the delivery of PM, as well as to identify barriers and challenges that influence provision of PM, along with innovative practices implemented to facilitate provision. While the application of PM is growing continuously, its use is currently most advanced in oncology (Brittain et al., 2017; Jackson & Chester, 2015). Therefore, the review will focus on HCPs delivering PM for the treatment of cancer.

## Methods

A scoping review was undertaken, adopting the methodological framework developed by Arksey and O’Malley and Levac et al. (Arksey & O’Malley, 2005; Levac et al., 2010). The PRISMA-ScR (Preferred Reporting Items for Systematic Reviews and Meta-Analysis extension for Scoping Reviews) checklist was followed for reporting purposes (Tricco et al., 2018). A study protocol was developed and registered through the Open Science Framework (https://doi.org/10.17605/OSF.IO/W9YTR). This study aimed to address the following research questions:

- What are the knowledge of and attitudes toward, and adoption practices around HCPs in PM for cancer?
- What barriers and challenges are faced by HCPs in providing PM therapies for cancer?
- What innovative practices are employed for the provision of PM?

### Database search strategy

An extensive search for relevant articles was conducted in PubMed, CINAHL, Embase, Scopus, Web of Science and ProQuest on 02/12/2022. The search strategy was developed using the PCC framework; population (HCPs), concept (PM) and context (cancer); as guided by the Joanna Briggs Institute Reviewer’s Manual, 2015 (Peters et al., 2015). Medical Subject Headings (MeSH) and synonyms of PM were also included in the search strategy to accommodate the fluidity and polysemy in the concept of PM (Ali-Khan et al., 2016; De Grandis & Halgunset, 2016). For the population, the search terms were limited to oncology team members involved in provision of PM (nurse, primary care physician, pharmacist, and oncologist). The details of the terms used for the search strategy are described in Table 1 while the search ran in PubMed is described in the supplementary material. Preliminary research indicated that the first PM approved for cancer was Keytruda in 2014 (Raedler, 2015). Hence, the time limit for the database search was restricted to 2014–2022. All results were exported to and managed in Endnote X9 (Clarivate Analytics, PA, USA). The articles identified through the database search were uploaded to Rayyan (http://rayyan.qcri.org/) after de-duplication.

**Table 1.** Population, concept and context (PCC) framework used for searching of databases.

Population	Concept	Context
Health personnel, health care personnel, healthcare personnel, health care provider, healthcare provider, health provider, healthcare worker, health worker, health care worker, health care professional, healthcare professional, health profession personnel, health care practitioner, healthcare practitioner, health practitioner, medical personnel Primary care physicians, physician*, nurse*, doctor*, pharmacist*, oncologist*, general practitioner*, GP, family physician	Precision medicine, genomic medicine, genetic medicine, pharmacogenomic, pharmacogenetic, personalised medicine, personalized medicine, individualised medicine, individualized medicine, genetic screening, genetic testing, predictive medicine, precision cancer medicine, precision therapy, individualised therapy, individualized therapy, personalised therapy, personalized therapy, cell and gene therapy, stratified medicine, gene therapy, Genetic therapy	Neoplasms, tumor, tumour cancer, malignancy*, malignant neoplasm, malignant neoplastic disease, neoplasia

### Inclusion/exclusion criteria

Only studies published in English and addressing knowledge, attitude and practice of HCPs towards PM for cancer; barriers and facilitators faced by HCPs in providing PM; and innovative practices employed for the provision of PM, were included in this review. Articles were excluded if the concept did not align with PM or its various synonyms; referred to any disease other than cancer; were commentaries, abstracts, editorials, opinion pieces; study protocols; or research on animals.

### Study selection

The study selection process involved title and abstract screening, and full-text screening. Two researchers (SS and GW) screened the title and abstracts of a randomly chosen sample of items (10% of all) and discussed arising uncertainties. The procedure was repeated until the researchers achieved a 90% agreement of decisions in the given sample. Following this step, the remaining articles were screened by one researcher based on the eligibility criteria and agreed decisions. The full text of the short-listed studies was then assessed for eligibility by two researchers (SS and GW). Any conflicts at this stage were arbitrated by the lead researcher (SS) or by a third researcher (SG) and resolved.

### Data charting

A data charting form was designed in Microsoft Excel and tested by the research team before data charting. Two researchers (SS and GW) independently charted data for a random 10% of the studies and discussed them. The procedure was repeated until 90% consistency was reached on data charting procedures. The data for the remaining studies were charted by one researcher (SS or GW). Data were charted for the following: author’s name, title, study location, objective, terminology used, study population and key findings.

### Data analysis

The quantitative data were described using descriptive statistics. The qualitative data charted were analysed thematically, following three of the six steps described by Braun and Clarke: familiarisation with the data, generation of codes and production of a report (Braun & Clarke, 2006). SS first read the charted data to become familiar with the content and sorted the charted information by research question. Using an open coding technique, SS developed the codes in Microsoft Excel. As the data was charted in Excel, open codes were also developed in Excel through systematic colour coding and highlighting of texts. Once the initial codes were generated, they were organised into broad themes and sub-themes. The codes and themes were discussed with GW and SG to ensure that they were mutually exclusive and collectively comprehensive. The analysed data was then synthesised narratively.

## Results

**Figure 1.**
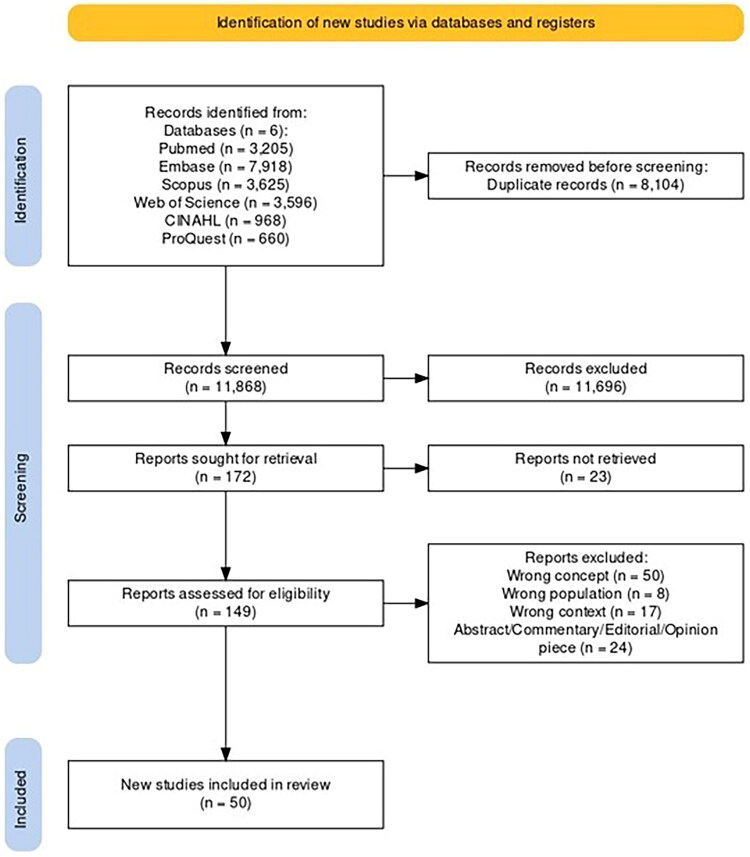
PRISMA flowchart for the scoping review (Created using PRISMA2020: An R package and Shiny app for producing PRISMA 2020-compliant flow diagrams, with interactivity for optimised digital transparency and Open Synthesis Campbell Systematic Reviews).

A total of 19,972 articles were identified that referenced PM. After the removal of 8104 duplicates, 11,868 were retained and the titles and abstracts of these articles were screened. Following this screening, 149 articles were included for the full-text review, which left 50 articles that were included in the final review (Figure 1).

### Characteristics of the included studies

**Figure 2.**
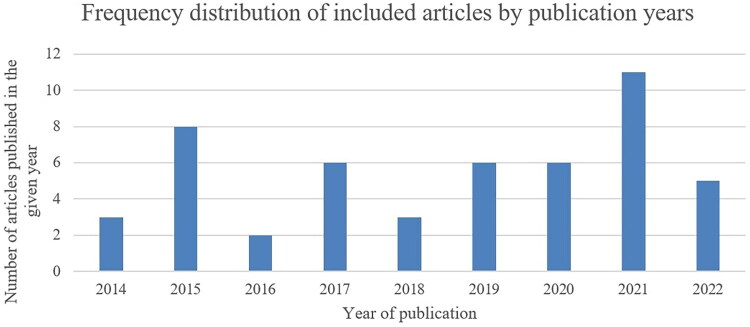
Frequency distribution of the included studies by the year of publication.

The characteristics of the studies included in this review are summarised in Table 2. Most of the studies were conducted in North America (*n* = 22) while three were conducted in Africa. There was variation in the terminologies used across the included studies, with commonly used terms being genetic testing (*n* = 11), precision medicine (*n* = 6), pharmacogenomics (*n* = 6), and personalised medicine (*n* = 4). Most of the studies were primary studies employing surveys (*n* = 22), and interview methods (*n* = 7). As shown in Figure 2, most of the articles were published in 2021 (*n* = 11), followed by 2015 (*n* = 8).

**Table 2.** Characteristics of the included studies.

Author	Country	‘Objective’	Terminology used	Methods	Study population
Adejumo et al. (2021)	Nigeria	To evaluate university nursing students’ knowledge of genomic concepts and readiness to practice and genomic nursing in Nigeria	Genomic nursing	Survey	Nursing students
Ademuyiwa et al. (2021)	USA	To understand US breast oncology physicians knowledge, attitudes and practices regarding genetic counselling and testing (GCT) in African American women with breast cancer	Genetic counselling and testing	Survey	Oncology physicians
Adeniji et al. (2021)	Nigeria, Nepal	To identify relevant barriers and challenges that limit the implementation of personalised medicine in the developing world with an emphasis on Nigeria and Nepal	Personalised medicine	Narrative review	N/R
Al Bakir et al. (2019)	UK	To capture the current state of genomics training in gastroenterology to review current understanding, clinical experience and long-term educational needs of UK trainees.	Genomic medicine	Survey	Specialty trainees
Albitar and Abou Alchamat (2021)	Syria	To evaluate Syrian pharmacists and physicians knowledge in pharmacogenetics and the attitude towards it	Pharmacogenetics	Survey	Physicians, pharmacists and academics
Anderson et al. (2021)	USA	To understand the perception of community oncology clinicians towards genomic tumour testing (GTT) and how they relate to clinicians’ intentions to use GTT.	Genomic tumour testing	Survey	Oncology physicians
Aomori et al. (2022)	Japan	To highlight the cancer genomic medicine (CGM) system in Japan, the issues it faces, and the role of pharmacists in this system	Cancer genomic medicine (CGM)	Narrative review	Pharmacists
Arnall et al. (2019)	USA	To describe the implementation, benefits, and challenges of clinical pharmacist services within a Precision Medicine Program for cancer patients	Precision medicine	Narrative review	Pharmacy resident
Bazarbashi et al. (2022)	Middle East and Africa	To identify the real-world challenges in diagnostics and treatment of Metastatic Castration-Resistant Prostate Cancer (mCRPC) and provide insights on the urgent unmet needs	Genetic testing	Consensus discussion	Oncologist (medical oncologist, clinical oncologists) and professors
Bokkers et al. (2022)	Netherlands	To assess health care professionals’ attitudes toward and knowledge of mainstream genetic testing, and their self-efficacy to discuss genetic testing before and 6 months after completion of a training module; To perform user evaluation of the training modules; To gain insight into the feasibility for health care professionals to incorporate mainstream genetic testing into the routine care of women with epithelial ovarian cancer (EOC).	Genetic testing	Survey	Healthcare professionals
Bol and Meric-Bernstam (2015)	USA	To describe the role of surgeons in building a personalised medicine programme	Personalised medicine	Narrative review	Surgeons
Bombard et al. (2015)	Canada	To explore medical oncologists’ views of gene expression profiling tests and factors impacting its use in clinical practice.	Gene expression profiling tests	Interview	Oncologists
Borden et al. (2019)	USA	To study provider attitudes of and perceived barriers to the clinical use of phamacogenomics before and during participation in an implementation programme	Pharmacogenomics	Interview and survey	Health Care Providers
Calzone et al. (2018)	USA	To assess leadership team (administrator/educator) year-long interventions to improve registered nurses (RNs) capacity to integrate genomics into practice	Genomics	Survey	Registered nurses
Caraballo et al. (2017)	USA	To describe a comprehensive and systematic implementation model to overcome the challenges faced in the implementation of pharmacogenomics	Pharmacogenomics	Evaluation of outcomes	N/R
Carroll et al. (2019)	Canada	To determine family physicians’ (FP) current involvement in genomic medicine (GM), confidence in GM primary care competencies, attitudes regarding the clinical importance of GM, awareness of genetic services, resources required, and suggestions for changes that would enable integration of GM into practice	Genomic medicine	Survey	Family physicians
Cho et al. (2020)	South Korea	To investigate awareness, attitudes and perspectives on precision medicine among health professionals in South Korea and to identify issues that need to be addressed before implementing precision medicine	Precision medicine	Interview and survey	Healthcare professionals
Ciardiello et al. (2016)	International	To explore self-reported and physician-assessed levels of patient cancer literacy and factors affecting physicians’ choice to use biomarkers in treatment decisions	Precision medicine	Interview	Physicians
Cusack et al. (2021)	Australia	To inform continuing education of general practitioners (GPs) in Australia by identifying GPs’ views on genomics, the impact of genomics on their practice, and their educational needs.	Genomics	Interview	General practitioners
De Abreu Lourenco et al. (2021)	Australia and New Zealand	To investigate the factors that influence decision making in genomic medicine from the perspective of different stakeholders in the context of difficult-to-treat childhood cancer	Genomic medicine	Survey	Oncology medical, nursing and allied health staff, clinical researchers and affiliated specialist staff directly involved in provision of paediatric cancer care.
Delikurt et al. (2015)	International	To identify factors that have impact on access to genetic services for patients by influencing referral; To enhance understanding of how patient-related access to genetic services has been measured thus far in the current literature; To identify similarities and differences, if any, in the published research reporting evidence on barriers to patients’ access.	Genetic services	Systematic review	Health care professionals
Diamonstein et al. (2018)	USA	To evaluate practicing physicians’ awareness of, utilisation of and perceived barriers to genetic services in Texas, and interest in learning more about genetics and genetic services	Genetics and genetic services	Survey	Physicians
Dias et al. (2014)	Australia	To understand the perceptions, barriers and drivers of the practice of pharmacogenomics among hospital pharmacists in South Australia	Pharmacogenomics	Interview	Pharmacists
Eum et al. (2018)	South Korea	To examine awareness of genetic testing in Korea; To assess difference in attitudes toward genetic testing among the general public, cancer patients and healthcare professionals; To suggest ways in which to improve and utilise knowledge of genetic testing for better patient care	Genetic testing	Survey	Clinicians, nurses
Fountzilas et al. (2022)	Greece	Evaluate the counselling practices and experiences before and after genetic testing for hereditary cancer in Greece and evaluate whether these results could provide critical areas for improvement of genetic counselling processes.	Genetic testing	Survey	Medical oncologists, gynecologists and general surgeons
Fu et al. (2020)	USA	To provide an overview of precision health and the importance of engaging the nursing profession for its implementation.	Precision medicine	Narrative review	NA
Hall et al. (2015)	USA	To understand patient and providers perception and expectation of genomic medicine	Genomic medicine	Narrative review	Healthcare providers
Hamilton et al. (2017)	USA	To identify studies of US primary care providers (PCPs’) knowledge, attitudes, and communication-related behaviours regarding genetic tests	Genetic testing	Systematic review	Primary care providers
Hamilton et al. (2021)	USA	To describe patient communication challenges encountered by oncology clinicians to implement precision oncology	Precision medicine	Focus group discussion	Oncology clinicians
Hann et al. (2017)	UK	To investigate UK health care professionals (HCPs’) knowledge of ovarian cancer genetics and other risk factors, as well as self-efficacy in discussing cancer risk and genetic testing with patients; To assess attitudes towards population-based genetic testing and stratified risk management strategies for ovarian cancer.	Genetic testing	Survey	Oncologists, genetics clinicians, general practitioners, gynaecologists, nurses
Harding et al. (2019)	Canada	To explore genetics in primary care from the perspective of both rural and urban PCPs	Genetics	Interview	Primary care providers
Hinderer et al. (2017)	Germany	To assess the physicians’ attitude, their knowledge and their experience in pharmacogenomic clinical decision support in German hospitals.	Pharmacogenomics	Survey	Physicians
Kolesar and Vermeulen (2021)	USA	To describe current pharmacist roles in genomic aspects of precision medicine, to assess barriers and facilitators to implementing precision medicine, and to discuss emerging trends likely to impact health systems.	Precision medicine	Narrative review	Pharmacist
Komatsu and Yagasaki (2014)	Japan	To explore the recognition, implementation and challenges of hereditary breast and ovarian cancer (HBOC) risk assessment and management from the perspective of breast cancer providers and to explore the readiness for personalised cancer risk management at the level of clinical practice.	Personalised medicine	Focus group discussion	Breast cancer care providers
Li et al. (2015)	China	To explore the relationship between physicians knowledge and utilisation of genetic testing and to explore genetics educational needs in China	Genetic testing	Survey	Physicians
McAllister and Schmitt (2015)	USA	To develop a timely, evidence-based process for the use of Oncotype DX test results to enhance decision making for women with early-stage, ER+, HER2/neu-negative breast cancer.	Genomic testing	Evaluation of outcomes	Nurses
McCauley et al. (2017)	USA	To explore Wisconsin physicians’ views, practices and educational desires regarding genetic and genomic testing.	Genomic/genetic testing	Survey	Physicians
Nagy, Lynch, et al. (2020)	Egypt	To assess healthcare practitioners’ perspectives regarding clinical pharmacogenetics in Cairo, Egypt	Pharmacogenetics	Survey	Healthcare practitioners
Nagy, Tsermpini, et al. (2020)	Egypt	To identify the educational challenges for pharmacogenomics integration into clinical practice and their impact on pharmacists’ knowledge and confidence, in addition to underscoring pharmacists’ role in pharmacogenomics as a whole.	Pharmacogenomics	Narrative review	Pharmacists
Ong et al. (2022)	Singapore	To review existing literature on GP’s experience, attitudes and needs towards clinical genetic services.	Genetic services	Narrative review	General pracitioners
Pokharel et al. (2016)	Nepal	To explore knowledge, attitudes and perception of Nepalese physicians towards genetic testing for gynaecologic cancer	Genetic testing	Survey	General practitioners and specialists
Przybylski et al. (2020)	USA	To identify opportunities for health systems to increase the implementation and adoption of oncology focused pharmacogenomics services.	Pharmacogenomics	Survey	Pharmacist
Roberts et al. (2016)	USA	To better understand US oncologists oncotype DX (ODX) uptake and how they use ODX during adjuvant chemotherapy decision making	Genetic testing	Interview	Oncologist
Shelton and Whitcomb (2015)	USA	To describe the challenges, controversies, and opportunities for genetics and genetic counsellors in managing complex disorders and discuss the rationale for modifications in genetic counsellor training and function.	Personalised medicine	Narrative review	Physicians and genetic counsellors
Smit et al. (2021)	Australia, USA and Canada	To assess the knowledge, attitudes and expectations regarding polygenic testing among health professionals who provide cancer risk assessments.	Polygenic testing	Survey	Health professionals
Teng and Spigelman (2014)	Australia	To assess doctors’ referral rates, knowledge and attitudes towards cancer genetic testing	Genetic testing	Survey	GPs and specialists
Thavaneswaran et al. (2021)	Australia	To characterise oncologists experiences and needs when utilising genomic results	Comprehensive genomic profiling	Survey	Oncologists
Vashistha et al. (2020)	USA	To assess oncologists’ practices, concerns, and perceptions regarding Next-Generation Sequencing (NGS) and the National precision oncology program (NPOP)	Precision oncology	Interview	Oncologists
Vetsch et al. (2019)	Australia	To explore the existing literature on health care professionals (HCPs’) attitudes towards cancer precision medicine	Cancer precision medicine	Systematic review	HCPs
Wevers et al. (2017)	Netherlands	To assess the knowledge and attitudes of professionals towards rapid genetic counselling and testing (RGCT)	Rapid genetic counselling and testing	Evaluation of outcomes	Health professionals

### Knowledge, attitudes and adoption practices of HCPs towards PM

Thirty-two studies assessed some aspects of knowledge, attitudes or perceptions related to PM as shown in Tables 3 and 4. Overall, we observed wide variation in the reported knowledge, attitudes and confidence of HCPs towards PM. Two of the studies reported that HCPs had good knowledge of PM (Ademuyiwa et al., 2021; Hann et al., 2017). Almost all the participants (97.1%) indicated having overall good knowledge in the US-based study by Ademuyiwa et al. (2021). On the contrary, three studies, conducted at around the same time in Nigeria, Syria and Germany, reported poor knowledge among the majority of study participants (49.6–89%) (Adejumo et al., 2021; Albitar & Abou Alchamat, 2021; Hinderer et al., 2017). One study reported little or no awareness of genetic testing among 21% of general practitioners (Diamonstein et al., 2018). In the same vein, one study noted that general practitioners had the worst knowledge level compared to other specialists such as breast/ovarian cancer specialist or, gastrointestinal specialist (Teng & Spigelman, 2014).

**Table 3.** Knowledge, attitudes and perceptions of healthcare personnel (HCP) towards personalised medicine [quantitative findings].

Author	Tool	Measurement	Quantitative findings
Knowledge, awareness and experience			
Adejumo et al. (2021)	Adapted genetic nursing concept inventory questionnaire	60 items to assess participants’ knowledge, with 1 point being assigned to each correct answer. Scores below 30 were categorised as poor knowledge while scores ≥30 were categorised as good knowledge	89% had poor knowledge. 11% scored more than 50% knowledge score. Mean knowledge score: 16.6 ± 8
Ademuyiwa et al. (2021)	Survey	49-item survey	97.1% had good knowledge and 73.3% had a good understanding of breast and ovarian cancer genetics.
Albitar and Abou Alchamat (2021)	Survey	18 questions assessing knowledge of pharmacogenetics	63.1% declared that they did not have sufficient knowledge of pharmacogenetics testing. Significant association between pharmacogenetics testing and knowledge (*p* = 0.005) Significant association between profession and pharmacogenetics knowledge (*p* = 0.049). Respondents of younger age and less experienced professionals had the highest knowledge of pharmacogenetics.
Carroll et al. (2019)	Survey	12 questions assessing awareness of and experience with genetic services 18 questions assessing knowledge	22.3% agreed that they could identify useful sources of information regarding genetics for their practice. 21.3% could find information about genetic tests available within the healthcare system. Median knowledge score on the 10 clinical vignettes was 6/10 (range: 0 to 10). 31% indicated that they were unaware of the answer.
Diamonstein et al. (2018)	Survey	8 items on awareness of genetic services	21% and 31% reported little or no awareness of genetic testing and services respectively. 50% and 47% reported awareness of genetic testing and services respectively.
Hann et al. (2017)	Survey	Knowledge assessed using five True/ false/ Not sure questions and three multiple choice questions. Correctly answered questions were given a score of 0–8.	Participants had a high knowledge of ovarian cancer and related genetics (Median score: 7 out of 8, Interquartile range: 3.0). The knowledge score of general physicians’ (Media: 4) was significantly lower compared to genetic clinicians (Median: 8, *p* < 0.001), oncologists (Median: 7, *p* < 0.001) and gynaecologists (Median = 7.0, *p* < 0.001).
Hinderer et al. (2017)	Survey	5 items measuring self-reported knowledge of genetics and genomics	49.6% reported a deficit in their knowledge of genetics, while only 50.5% physicians reported their knowledge being nearly equally good.
Li et al. (2015)	Survey	Self-rate knowledge in about six categories of genetic testing techniques	The average personal genetic knowledge score was 2.1 ± 0.8. There was a huge gap between Chinese physician’s knowledge and utilisation of genetic testing. Physicians with more genetic knowledge were more confident integrating new genetic testing techniques into their practice.
McCauley et al. (2017)	Survey	Questions phrased as dichotomous (Yes/No) questions or as Likert-scale items	Few physicians had significant experience or felt prepared to use genetic tests.
Nagy, Lynch, et al. (2020)	Survey	6 questions assessing knowledge, out of which 5 were fact-based question. Each correct answer was assigned 1 point, while incorrect or not sure were allocated zero points. The total score was divided by 5 and multiplied by 100 to get participant’s ‘knowledge score’.	The mean knowledge score was 41.7 ± 21.5%. Physicians and pharmacists with previous pharmacogenetic training or education had higher knowledge scores (47.5 ± 22.9% vs 41.2 ± 21.3%). However, the results were not statistically significant (*p* = 0.26).
Teng and Spigelman (2014)	Survey	Correct answers to 5 knowledge questions were measured.	Doctors had suboptimal knowledge of cancer genetic testing. General Practitioners (GPs) had the worst knowledge while breast/ovarian specialists were the most knowledgeable; followed by gastrointestinal specialists and other specialists.
Attitudes and perceptions			
Albitar and Abou Alchamat (2021)	Internet based survey	4 questions assessing personal attitudes towards pharmacogenetics	Most of the respondents (42.9%) agreed that pharmacogenetics should be a priority in college education.
Anderson et al. (2021)	Survey	9 attitude questions	A positive attitude towards the value of GTT (Mean attitude score: 2.48 ± 0.46).
Carroll et al. (2019)	Survey	11 questions assessing attitudes towards genomic medicine	Mixed attitudes related to genomic medicine (OR: 2.44; 95% CI: 1.24–4.80; *p* = 0.010). Only 59.4% of the respondents showed agreement or strong agreement to advances in genomic medicine bringing about improvement in patients’ health outcomes. Only 43.1% agreed on the importance of learning about personalised patient care based on targeted or whole genome sequencing and less than half (36.3%) agreed that it was their responsibility to incorporate genomic medicine into their practice.
Cho et al. (2020)	Survey	NR	96% agreed that precision medicine would be effective in patient treatment. 94.9% agreed that precision medicine would provide precise diagnosis.
Ciardiello et al. (2016)	Survey	NR	Majority of the physicians (82%) believed that the treatment decision was a shared decision-making process among the doctor, multi-disciplinary team and patient. Comparatively less proportion of respondents in Saudi Arabia held this belief (21%) compared to more than 97% of the respondents in Brazil, China and Turkey who agreed with the multi-disciplinary team approach. The consensus on multi-disciplinary team was also comparatively low in Spain, Argentina and Russia (72%, 77%, and 70%, respectively).
Diamonstein et al. (2018)	Survey	4 items assessing general perception of genetics in medicine	42% of the respondents felt that genetics was moderately or very integral part of patient care within their specialty. Participants specialising in Ob/ Gyn (92%) and paediatrics (73%), perceived genetics as more integral to their patient care than other (24%) specialties and also reported discussing genetics more in their day-to-day practice.
Eum et al. (2018)	Survey	8 questions assessing attitudes towards genetic testing	30.1% of the clinicians showed agreement to inclusion of genetic testing in the national screening programme. 70.8% of clinicians believed that people had the right to know about their genes so that they could take actions for their health. Clinicians strongly believed that knowledge of test results could lead to discrimination, compared to the other sample groups (Clinicians: 87.6%; public: 70.7%; patients: 68.8%; and researchers: 81.4%).
Hann et al. (2017)	Survey	7-items assessing attitudes using a 5-point Likert scale	There was mixed attitude towards population-based genetic testing for ovarian cancer risk. Most of the HCPs acknowledged the potential benefits of genetic testing for patients (Agreed: 71.2%; strongly agreed: 10.3%), while many also shared concerns about the negative impact on some patients (Agreed: 64.4%; strongly agreed: 9.6%). Nearly half of the HCPs believed that patients could be discriminated by the insurers based on the test results (Agreed: 43.2%; strongly agreed: 2.7%). 47.9% of the respondents showed willingness to offer all adult female patients genetic testing for ovarian cancer risk.
Li et al. (2015)	Survey	Self-perceived educational needs	84% participants shared a desire for additional genetic education.
Nagy, Lynch, et al. (2020)	Survey	8 questions assessing attitudes towards pharmacogenetics and its clinical implications, measured on a 5-point Likert scale.	Participants had a positive attitude with 68.5% of participants agreeing or strongly agreeing to the questions assessing attitude towards pharmacogenetics. Pharmacists showed more interest in pharmacogenetic training in the future as compared to physicians (64% vs 37.3%; *p* < 0.0001).
Thavaneswaran et al. (2021)	Survey	NR	97% of the oncologists perceived that it was their responsibility to inform the patients about comprehensive genomic profiling (CGP). More than half of the oncologists (63%) wanted support to translate genomic information into recommendations for treatment.
Wevers et al. (2017)	Questionnaire	Questionnaire filled at baseline, 6 months and 12 months follow up	44% of surgeons and 33% of specialised nurses indicated that Rapid Genetic Counselling and Testing (RGCT) was burdensome for patients. Most of the surgeons (94% before and after the study) and all of the specialised nurses believed that it was important to have the option to refer patients for RGCT. At the end of the study, most of the health professionals believed that the advantages of RGCT outweigh the disadvantages.
Confidence			
Anderson et al. (2021)	Survey questionnaire	Confidence measured through internal confidence (clinicians’ confidence or self-efficacy regarding their own ability to use genomic tumour testing (GTT)) and external confidence (clinicians’ confidence in the ability of other stakeholders to use GTT). Clinicians were asked to rate their confidence.	High but variable level of confidence. Mean internal confidence: 2.62 ± 0.75, Mean external confidence score: 2.18 ± 0.65.
Carroll et al. (2019)	Survey	14 questions assessing confidence in the tasks of each role providing genetic services in their practice	Family physicians had low self-reported confidence in performing tasks related to the delivery of traditional genomic medicine. Even for tasks requiring high involvement skills, the confidence was found to be moderate (ranging from 21.3% to 55.3%). Respondents with continuing education in genetics in the past five years had significantly higher confidence in a range of genomic medicine skills.
Smit et al. (2021)	Online questionnaire	Measured using six items for which participants indicated their level of confidence (1 = not confident at all, 4 = very confident)	The study participants had low confidence in implementing findings from polygenic cancer testing in a clinical setting. Participants also had low confidence in discussing insurance implications, recommending test for a patient who is at risk of cancer and interpreting results from polygenic testing. Although, participants were confident in starting conversations around polygenic testing. About 28% of the participants were unsure about the potential integration of polygenic testing into their clinical practice and only 9.5% and 1% felt adequately and very prepared for the integration respectively.
Readiness to practice personalised medicine			
Adejumo et al. (2021)	Adapted genetic nursing concept inventory questionnaire	10 items to assess participants’ readiness to integrate genomic knowledge into practice measured on a 5-point Likert scale.	Not ready to practice genetic nursing in the future: 66% Mean readiness score: 18.5 ± 13 40% denoted satisfaction towards practicing genomic nursing in the future.
Al Bakir et al. (2019)	Web-based survey comprising of 12 questions	NR	Less than 40% felt clinically prepared to practice genomic medicine.
Przybylski et al. (2020)	Survey	Pharmacists were assessed on their comfort level with 5 variables. They were classified as comfortable if they were comfortable with 3 or more variables. Those comfortable with 2 or less of these variables were classified as not comfortable.	Oncology pharmacists with more than 10 years of experience were more likely to be comfortable in making assessments of pharmacogenomic data (*p* = 0.02). Pharmacists with a post-graduate education (Post-graduate year 2) or fellowship training in oncology showed greater comfort in assessing pharmacogenomic results (*p* = 0.04). The comfort level of pharmacists was higher if the institution’s MTB comprised of a pharmacist (*p* = 0.01) or had local pharmacogenomics policies (*p* = 0.02).
Education and training			
Al Bakir et al. (2019)	Web-based survey comprising of 12 questions	NR	Only 9% and 16% of survey respondents believed that their local training programme was sufficed for them to use genomic medicine and personalised medicine. Majority of the respondents (95%) showed agreement to a need of more education before mainstreaming genetic testing.
Diamonstein et al. (2018)	Survey	2 items assessing interest in learning about genetics and genetic testing	Majority of the respondents were keen on learning about genetics and genetic testing and genetic services available to their patients (77% and 78% respectively).
Hinderer et al. (2017)	Survey	4 items assessing education and training in pharmacogenomics testing	80.4% believed that training on pharmacogenomics tests was required for them.
McCauley et al. (2017)	Survey	13 items assessing perceived learning needs	As compared to younger physicians (18.6%), adult physicians were less likely to feel adequacy in their training in genetics/ genomics (10.4%). Most physicians had a keen interest in furthering their genetics/ genomics education (Adult primary care physicians: 80.6%; all other physicians: 78.4%).
Teng and Spigelman (2014)	Survey	NR	77.6% of doctors showed a desire for more information on cancer genetic testing. Certain specialists like breast/ovarian specialists (33.3%) and ‘other’ specialists (25%) were the least interested in getting further information about cancer genetic testing compared to gastrointestinal specialists (90%) and GPs (84%). The difference between the groups was significant (*p* < 0.005).
Utilisation			
Borden et al. (2019)	Survey	A base line questionnaire which was filled at baseline and also filled by the participants every 3 months throughout the study period.	Forty-two percent of the providers were likely or influenced by the delivered pharmacogenomic results.
Ciardiello et al. (2016)	Interview survey	NR	Most of the physicians (90%) reported using biomarkers.
Hinderer et al. (2017)	Survey	7 items assessing use of pharmacogenomics clinical decision support system (CDSS)	97.5% believed that a pharmacogenomic CDSS would be useful for clinical tasks and showed a keen interest in using such systems.

N/R: Not Reported.

**Table 4.** Knowledge, attitudes and perceptions of healthcare personnel (HCP) towards personalised medicine [qualitative findings].

Author	Qualitative findings
Bombard et al. (2015)	There were mixed attitudes among the oncologists about gene expression profiling (GEP). GEP enhanced the confidence of some oncologists in their risk assessment approach while others considered GEP to be critical in resolving uncertainties related to chemotherapy recommendations.
Cho et al. (2020)	Majority of the respondents (95–96%) agreed that precision medicine would be effective in treatment and precise diagnosis. However, most of the health professionals were not confident on the patient’s ability to understand precision medicine and shared that lack of competencies among physicians could be an issue. In the focus group interview, respondents suggested that most of the clinicians were not ready to provide genetic testing, interpretation, and personalised treatment to their patients.
Cusack et al. (2021)	The respondents had a positive perception on the impact of genomic medicine in healthcare and its future role. However, many participants lacked confidence in understanding the different nuances between genomic testing and genetic testing. The participants showed keen interest in learning about genomics relevant to their practice.
De Abreu Lourenco et al. (2021)	The participants (HCPs, parents and the general community) were more likely to recommend or participate in genomically guided treatment if it was publicly funded and less likely to participate or recommend if parents were asked to pay. All study participant groups were more likely to choose to recommend/participate in a genomically guided approach if it was also supported by the decisional partner (e.g. the parents for HCPs or the converse).
Dias et al. (2014)	Pharmacists had not been involved with pharmacogenomics (PG) testing. They rarely used genomic information to provide medication-related advice. Pharmacists showed minimal interest in incorporating PG in their practice and also shared that they lacked confidence in relation to PG.
Hall et al. (2015)	The review reported that provides had a generally positive attitude toward genetic testing, yet only had modest experience with and knowledge of clinical genetics and genomics.
Hamilton et al. (2017)	Primary care providers (PCPs) had low levels of genetic testing-related knowledge. Basic knowledge of genetic testing was stronger among younger, more recent medical graduates specialists and providers in academic medical centres. PCPs showed interest in additional genetics education, opportunities to develop skills to interpret genetic tests and maintaining genetic privacy and confidentiality.
Hamilton et al. (2021)	The clinicians in the study used a range of terms including tailored treatments, personalised medicine, individualised care, and genomics, to describe precision oncology. Some clinicians also considered precision medicine as more of a buzzword and a marketing term.
Harding et al. (2019)	Primary care providers (PCPs) expressed a need for additional clarification on the interpretation of genetic test results, clinical utility, cost-effectiveness, and communication strategies. PCPs comfort with genetic testing depended on personal training and experience. PCPs were also interested in information about current resources, tests and referral guidelines.
Komatsu and Yagasaki (2014)	The study participants shared hesitancy in involving themselves in sensitive genetic issues as they considered it to be a complex problem. The participants also showed sub-optimal readiness for personalised cancer risk management.
Ong et al. (2022)	The review reported that general practitioners’ (GPs) lacked clarity about their role in clinical genetics. GPs also felt that genetics tasks required specialists’ knowledge and the tasks were complex in nature.
Vashistha et al. (2020)	Oncologists in Veterans Health Administration (VHA) were keen on widening their foundation understanding of tumour DNA sequencing. The presence of a national programme such as the National Precision Oncology Programme (NPOP) boosted VHA oncologists confidence not only in the improvement of outcomes of their patients by offering such therapies but also in furthering research initiatives.

Four studies reported positive attitudes of HCPs towards PM (Anderson et al., 2021; Cusack et al., 2021; Hall et al., 2015; Nagy, Lynch, et al., 2020), while three studies reported mixed attitudes (Bombard et al., 2015; Carroll et al., 2019; Hann et al., 2017). Further quantitative details on attitudes and perception of HCPs can be found in the respective section of Table 3. One study associated more knowledge of PM with more confidence among HCPs (Li et al., 2015), while another study reported HCPs with more experience being more comfortable in delivering PM (*p* = 0.02) (Przybylski et al., 2020). Ciardiello et al. (2016) reported that 82% of the HCPs believed that the treatment decision in PM was a shared decision-making process; Adeniji et al. (2021) additionally noted that HCPs’ knowledge and confidence also influenced that of patients.

Two studies reported that knowledge and feeling of adequacy in training were stronger among the younger physicians compared to adult physicians (Hamilton et al., 2017; McCauley et al., 2017). Carroll et al. (2019) reported similar findings, arguing that family physicians with continuing education in genetics in the past five years had significantly higher confidence in a number of genomic medicine skills (OR: 2.44; 95% CI: 1.24–4.80; *p* = 0.010). HCPs further believed that patients could be discriminated based on their test results (Eum et al., 2018; Hann et al., 2017).

Four studies also found low confidence among HCPs in providing PM (Carroll et al., 2019; Cusack et al., 2021; Dias et al., 2014; Smit et al., 2021). Only one study reported high but variable levels of confidence among HCPs (mean internal and external confidence score: 2.62 ± 0.75 and 2.18 ± 0.65 respectively) (Anderson et al., 2021). One study stated that the presence of a National Precision Oncology Programme (NPOP) boosted HCPs’ confidence in not only providing access to therapies to patients but also conducting future research, while another study reported 42% of HCP’s being influenced by the delivered pharmacogenomics results (Borden et al., 2019; Vashistha et al., 2020).

Six studies reported that about 63–95% of HCPs showed a desire for education, support, or training on PM, with two further studies reporting this desire for development narratively (Al Bakir et al., 2019; Cusack et al., 2021; Diamonstein et al., 2018; Harding et al., 2019; Li et al., 2015; McCauley et al., 2017; Teng & Spigelman, 2014; Thavaneswaran et al., 2021). One study also found that HCPs were more likely to recommend patients for genomics guided treatment if the treatment was publicly funded and supported by a decisional partner (De Abreu Lourenco et al., 2021).

### Barriers and challenges faced by HCPs in providing PM therapies

35 studies reported barriers and challenges faced by HCPs while providing PM to cancer patients. These barriers are categorised as: financial (*n* = 22), human resources (*n* = 26), infrastructural (*n* = 16), organisational and structural (*n* = 11), and other barriers (*n* = 22). All reported barriers and challenges are described in Table 5 while this section reports the commonly reported barriers and challenges under each category for succinctness.

**Table 5.** Barriers and challenges in the provision of personalised medicine.

Author/year/country	Financial barriers	Human resources related	Infrastructural	Organisational and structural	Other barriers
Adejumo et al. (2021) [Nigeria]	Inadequate funding of genomic related research and curricula development	Lack of trained personnel	N/R	N/R	Social and environmental factors
Ademuyiwa et al. (2021) [USA]	N/R	Lack of diversity among oncologists	Logistical barriers in relation to limited availability of genetic counsellors	N/R	Increased education needs of patients on genomic counselling and testing Health inequities and racial barriers in genetic counselling and testing
Adeniji et al. (2021) [Nigeria and Nepal]	Lack of funds	Lack of needed expertise	Absence of advanced genetic testing facilities Physician resistance Lack of immediate availability of novel drugs in developing nations Clinical trials/precision medicine barriers in drug development		Patient unawareness
Al Bakir et al. (2019) [UK]	Cost-effectiveness of genomic testing	N/R	N/R	Inadequacy of legal protections against discrimination for those individuals with genetic susceptibilities to disease	Lack of clinical guidance to guide interventions based on the results of genetic testing
Albitar and Abou Alchamat (2021) [Syria]	Economic embargo and sanctions	N/R	Limited resources	Restricted genomic studies	N/R
Anderson et al. (2021) [USA]	Lack of insurance coverage	Lack of patient and colleague interest	N/R	N/R	Managing patient expectations, litigation, and patient privacy with variants of unknown significanceLow probability of finding actionable results
Aomori et al. (2022) [Japan]	Problem with costs	The attending physician may not always be familiar with the therapeutic agent	N/R	N/R	N/R
Bazarbashi et al. (2022) [Middle East and Africa]	Economic constraints Genetic testing not reimbursed by social security, government or private insruance	Limited access of genetic providers	Limited access to genetic testing	Majority of the workforce present in urban areas and academic institutions.	Long duration for obtaining results
Bol and Meric-Bernstam (2015) [USA]	N/R	Dilemma faced by clinicians on whom to offer molecular profiling and the clinical context in which it is the most beneficial	N/R	N/R	N/R
Bombard et al. (2015) [Canada]	Costs of gene expression profiling Proprietary nature of the technology and the associated high cost	N/R	Overuse and inappropriate use and over-reliance on the results within the medical community	N/R	Uncertainty about patients understanding of GEP tests and their treatment implications Aggressive marketing of the product
Borden et al. (2019) [USA]	Cost or insurance coverage regarding testing and extra time spent in clinic due to incorporation of results	Lack of knowledge on pharmacogenomics Lack of formal training	N/R	N/R	Concerns around pharmacogenomic implementation, clinical utility of pharmacogenomic data, evidence supporting pharmacogenomic relationships
Caraballo et al. (2017) [USA]	Reimbursement for genetic testing	Prescriber uncertainty about the clinical and financial benefits of genome guided therapy Clinical resistance to provide support	Lack of standardisation between different laboratories in reporting PGx nomenclature as well as genotype-phenotype interpretations	N/R	Ethical and legal concerns Complexity in implementation as the nature of discovering new clinically actionable variants increases Lack of clinical practice guidelines to implement pharmacogenomics testing
Cho et al. (2020) [South Korea]	High cost of precision medicine	Challenges to train health workers and educating the public	N/R	Challenges in standardised data use	Precision medicine could increase disparity and health care costs.
Ciardiello et al. (2016) [International]	Cost of testing	N/R	Lack of local availability and speed of obtaining results	N/R	N/R
Cusack et al. (2021) [Australia]	N/R	Challenge in keeping up to date with the information	Challenges in managing large volume of genetic information through their generalist practice	Longer consultation time to explain limitations of the test and implications to the patient	N/R
Delikurt et al. (2015) [International]	N/R	Lack of knowledge of genetics and genetic conditions Lack of awareness of genetic services Lack of genetics workforce Lack of awareness of patient risk factors	N/R	Inadequate coordination of referral	Failure to obtain adequate family history
Diamonstein et al. (2018) [USA]	N/R	Lack of awareness on the availability of genetic counsellors Lack of understanding on when it is appropriate to refer genetic counsellor and lack of understanding on how to refer to a genetic counsellor	N/R	N/R	N/R
Dias et al. (2014) [Australia]	N/R	Pharmacists face difficulties to take on a pharmacogenomic role because of time constraints and occupation with other pharmacy activities Lack of timely and relevant pharmacogenomics education and information for pharmacists		N/R	N/R
Eum et al. (2018) [South Korea]	N/R	Limited physician education Lack of trained genetic counsellors	Lack of resources	Lack of genetics/genetic counselling departments in the hospitals	N/R
Fountzilas et al. (2022) [Greece]	N/R	Moderate/lack of educational training	N/R	N/R	Limited time availability
Hamilton et al. (2021) [USA]	Insurance related barriers and high co-payments	N/R	Logistical challenges of relevant tests, treatments and clinical trials	Organisational and structural challenges including lack of time	Lack of access particularly for patients in rural or underserved areas High expectations that patients hold for treatment benefits
Harding et al. (2019) [Canada]	Costs to patients for community based genetic testing	Limited access to staff Limited awareness about management options Lack of confidence in their genetic knowledge	Limited incorporation of foundational genetic education in undergraduate and post-graduate medical curricula Time, transportation, finances and missed employment were deterrents	Barriers in access to timely communication from referrals about follow-up plans especially for rural PCPs Limited access to ip-to-date materials	Lack of consensus about roles and responsibilities for genetics care
Hinderer et al. (2017) [Germany]	N/R	Lack of awareness about pharmacogenomic CDSS among physicians	N/R	N/R	N/R
Kolesar and Vermeulen (2021) [USA]	Poor reimbursement	N/R	Logistical challenges around testing	Lack of provider and administration buy-in	N/R
Komatsu and Yagasaki (2014) [Japan]	Lack of insurance coverage for genetic testing	N/R	Fragmented communication systems Limited resources	N/R	Ambiguity over responsibilities Legislations around sharing of genetic information within the multidisciplinary team
McCauley et al. (2017) [USA]	N/R	Physicians face barriers accessing training due to lack of time as a result of a busy clinical practice Lack of motivation to engage in genomics training given competing medical education Lack of basic understanding or awareness regarding genetic/genomic testing	N/R	N/R	N/R
Nagy, Lynch, et al. (2020) [Egypt]	Lack of funding and reimbursement Country’s economic status could affect the priority of perceived pharmacogenomics barriers	Lack of knowledge and confidence in pharmacists Lack of knowledge and training Insufficiency of trained personnel Limited pharmacogenomics education of undergraduate pharmacy students	Lack of required instruments for genotyping	N/R	Lack of pharmacogenomics guidelines
Nagy, Tsermpini, et al. (2020) [Egypt]	Limited funding	Lack of pharmacogenetic knowledge and skill Shortage of qualified personnel	Lack of pharmacogenetic testing devices	N/R	Lack of clinical guidelines Lack of time to adopt the application Patient refusal
Ong et al. (2022) [Singapore]	Limited reimbursement for GPs	N/R	N/R	N/R	Lack of clinical practice guidelines Clinical barriers such as rarity of cases, patients psychological well-being and concerns over the accuracy of genetic results Inaccuracies and gaps in information obtained from patient about their family history Time pressure faced by GPs
Pokharel et al. (2016) [Nepal]	Cost of testing	Lack of physicians information	Limited access to testing	N/R	Potential for increased patient anxiety Misinterpretation of results by patients Maintaining confidentiality of results
Przybylski et al. (2020) [USA]	Insurance denials of pharmacogenomic driven medications	N/R	N/R	N/R	Lack of clinical decision support tools to assist clinicians application of pharmacogenomic information Prolonged turnaround time of genetic results Visibility of pharmacogenomic results within the electronic medical record
Roberts et al. (2016) [USA]	N/R	Oncologists frequently faced difficulty communicating the purpose of ODX testing to patients	N/R	Organisational factors such as departmental structure, workflows for ordering tests and insurance policies	N/R
Shelton and Whitcomb (2015) [USA]	N/R	Lack of sufficient genetic counsellors or medical geneticists	N/R	N/R	N/R
Thavaneswaran et al. (2021) [Australia]	N/R	Lack of expertise to interpret the clinical finding	N/R	N/R	Uncertainty about the clinical relevance of the finding
Vetsch et al. (2019) [Australia]	Lack of public funding Lack of health insurance Limited opportunities to reimburse extra costs for time spent with patients and testing procedure	Limited knowledge and confidence of HCPs interpreting somatic test results	N/R	N/R	Concerns around inappropriate use of somatic testing to non-validated groups, over-reliance on test results

N/R: Not Reported.

The most frequently reported financial barrier was lack of or limited reimbursement mechanisms (*n* = 6) (Bazarbashi et al., 2022; Caraballo et al., 2017; Kolesar & Vermeulen, 2021; Nagy, Lynch, et al., 2020; Ong et al., 2022; Vetsch et al., 2019), lack of or limited insurance coverage including high co-pay (*n* = 5) (Anderson et al., 2021; Borden et al., 2019; Hamilton et al., 2021; Komatsu & Yagasaki, 2014; Przybylski et al., 2020), and the high cost of PM (*n* = 5) (Bombard et al., 2015; Cho et al., 2020; Ciardiello et al., 2016; Harding et al., 2019; Pokharel et al., 2016).

From a human resource perspective, the most significant barriers were: insufficient education and training, lack of knowledge, lack of familiarity of HCPs with PM, reported by 10 studies (Aomori et al., 2022; Borden et al., 2019; Delikurt et al., 2015; Dias et al., 2014; Fountzilas et al., 2022; Hinderer et al., 2017; Nagy, Lynch, et al., 2020; Nagy, Tsermpini, et al., 2020; Pokharel et al., 2016; Vetsch et al., 2019), and lack of trained personnel to provide PM, reported by a further seven (Adejumo et al., 2021; Adeniji et al., 2021; Delikurt et al., 2015; Eum et al., 2018; Nagy, Tsermpini, et al., 2020; Shelton & Whitcomb, 2015; Thavaneswaran et al., 2021). Other less commonly reported human resources barriers were: fear of discrimination for patients based on genetic test results (*n* = 1) (Al Bakir et al., 2019), lack of interest or motivation among colleagues and patients (Anderson et al., 2021; McCauley et al., 2017), and lack of confidence among HCPs (Harding et al., 2019).

Lack of access to specific testing technologies and facilities (*n* = 3) (Adeniji et al., 2021; Nagy, Lynch, et al., 2020; Pokharel et al., 2016), logistical challenges in organising tests, treatments and clinical trials (*n* = 3) (Adeniji et al., 2021; Hamilton et al., 2021; Kolesar & Vermeulen, 2021), and lack of resources (*n* = 3) (Albitar & Abou Alchamat, 2021; Eum et al., 2018; Komatsu & Yagasaki, 2014) were the most prevalent infrastructural barriers.

Various organisational barriers were reported, including inadequate legal protections against discrimination for individuals with genetic susceptibilities (Al Bakir et al., 2019), challenges in standardised use of data (Cho et al., 2020), lack of genetics/genetic counselling departments in the hospitals (Eum et al., 2018), and urban centric focus of workforce distribution (Bazarbashi et al., 2022).

Other commonly reported barriers were lack of clinical decision support tools or clinical guidance (*n* = 5) (Al Bakir et al., 2019; Caraballo et al., 2017; Nagy, Lynch, et al., 2020; Nagy, Tsermpini, et al., 2020; Ong et al., 2022). Further barriers under this category are reported in Table 5 .

### Innovative practices employed for provision of PM

Eight papers described innovative ways to improve the engagement of HCPs in providing PM (Table 6). Three studies (Arnall et al., 2019; Bol & Meric-Bernstam, 2015; Fu et al., 2020) narratively described the innovation while five studies also evaluated the innovations (Bokkers et al., 2022; Borden et al., 2019; Calzone et al., 2018; Caraballo et al., 2017; McAllister & Schmitt, 2015).

**Table 6.** Innovative approaches in personalised medicine.

Author/year	Innovation	Findings
Arnall et al. (2019)	Innovation: Precision Medicine Programme A clinical pharmacist was integrated into the Precision Medicine programme. The pharmacist collaborated with speciality pharmacy and facilitated drug assistance and dispensing. The pharmacist provided care to 14 oncology patients who were receiving precision-based therapies.	The oncologists and patients readily accepted the inclusion of the pharmacist in the team. The incorporation of the pharmacist in the team assisted in ensuring optimisation of therapies, reconciliation of medication, provision of supportive care and research management.
Borden et al. (2019)	Innovation: Patient specific pharmacogenomic results At the point-of-care, providers were provided with patient specific pharmacogenomic results through interactive clinical decision support (CDS).	Providers accessed the patient specific results at 64% of the visits, and medication changes were influenced by pharmacogenomic information in 42% of the instances. Providers usually felt confident in the information provided in the CDS summaries. Providers also felt that 74% of the time, they had adequate time to assess the results presented by GPS, 46% of the time the providers felt that the information exactly suited their patients situation, and 56% of the time the providers felt that the information supported by strong scientific evidence.
Bokkers et al. (2022)	Innovation: Online training module Gynaecologic oncologists, gynaecologists with a subspecialty training in oncology and nurse specialists were provided with an online training module. These professionals also received a training manual with instructions and necessary forms after the training. These trained health care professionals (HCPs) discussed the possibility of germline genetic testing (BRCA1/2, RAD51C/D and BRIP1) and the implications for family members with all newly diagnosed women with epithelial ovarian cancer (EOC) (including fallopian tube and extra ovarian carcinomas) and women who had a personal history of EOC and had not been tested previously. Additionally, HCPs also completed a checklist for every woman indicating whether she required additional counselling at the department of genetics after receiving their test result.	There was a significant increase in the knowledge of HCPs after 6 months of the intervention (*p* = 0.058). After completing the online training module and getting 6 months of hands on-experience, the attitude and self-efficacy of HCP’s remained positive and high respectively.
Bol and Meric-Bernstam (2015)	Innovation: Multidisciplinary team The study highlighted the importance of a multidisciplinary team (MDT) for creating an institution-wide personalised medicine platform. MDT would rely on the skills of pathaologists, medical oncologists, data analysts and informaticians. MDT would be connected by surgeons.MDT can review large datasets and huge data and consensually make decisions about application of new technologies and new tests in a clinical setting.	N/R
Calzone et al. (2018)	Innovation: Educator and nursing administrator dyad Dyads were pairs of hospital administrator and educator opinion leader pairs. An educator and a nursing administrator as part of a dyad were trained in genomics, genomic resources and educational strategies followed by monthly supplemental education and peer support. The dyads developed action plans for their institution using their hospital-specific baseline Genetics and Genomics Nursing Practice Survey data. The Dyads were in a strategic position for stakeholder engagement (for example: Board of Directors, Medicine, Pharmacy) and identification of institution level solutions (for example: providing resources for nursing education, modifying electronic health records).	There was a statistically significant increment in awareness and intention to learn among the intervention group compared to the controls group (*p* = 0.001). The findings implied that leadership was crucial in staff and resources mobilisation and supporting infrastructures for the sustenance of a competency effort on an institutional basis. The findings also implied longer intervention and support strategies such as infrastructure and policies to achieve genomic competency.
Caraballo et al. (2017)	Innovation: Implementation model for pharmacogenomics (PGx) The PGx implementation model comprised of eight interrelated functional components: **Institutional leadership support** Pharmacogenomics governance (formation of multidisciplinary task force to oversee all aspects of implementation of PGx) Clinical approval (identification and participation of clinical champions) Laboratory results (coordinate standard definitions for genotypes and phenotypes among different laboratories, implemented electronic interfaces between the laboratory systems and the HER when possible, implemented a manual review and data entry process when electronic interfaces between laboratory systems was not feasible) Pharmacogenomics education (systemic approach to PGx education, education designed for clinicians as well as pharmacists) **Pharmacogenomics Knowledge** **CDS-HER implementation** Long-term maintenance (strategy to maintain and update the data, knowledge, interfaces and CDS-HER applications).	In the duration of the intervention, 18 out of 21 drug-gene interactions reviewed were implemented in the PGx-CDS interventions. Thus, the model was concluded to be successful. In total, 11 educational resources to drug-gene interactions alongside 5 modules for pharmacists were developed and implemented. These resources were also welcomed by clinicians and pharmacists.
Fu et al. (2020)	Innovation: Engagement of nurses Nurses can be engaged in pharmacogenetics and pharmacogenomics to identify modification in human responses to pharmacological agents and diet. Nurses can further incorporate this knowledge into patient care and effectively monitor and manage care with pharmacological agents to improve and maintain the health of a patient.	The study recommended the following for effective translation and incorporation of precision health in future patient care: Funding to build the capacity of nurses to conduct research in precision health, develop their capacities through continuing education and training programmes Establish reimbursement from third party payers for precision health assessment Integration of precision health concepts and skills into nursing education Development of information content about precision health to empower the patients and the public.
McAllister and Schmitt (2015)	Innovation: Engagement of a navigator The study described the involvement of a navigator to assist and enhance decision making for women with early-stage ER+, HER2/neu-negative breast cancer while using Oncotype DX test results. A registered nurse with a bachelor’s degree fulfilled the role of the navigator. The nurse navigator worked with Advanced Practice Nurse (APN) to improve care for the patients.	The findings indicated that APN would be more suitable for the role of navigator. The APN could competently order the test and communicate the results to the patient and the medical oncologist. The introduction of the navigator led to a reduction in test ordering turnaround from 26.3 days to 11 days and also resulted in reduction of reporting turnaround from 38 days to 20 days. There was also an improvement in the compliance (from 26% to 88%) with the recommendations to perform Oncotype DX tests for eligible patients. The introduction of the navigator was considered to be successful and even led to a proposal to redesign the Registered Nurse Breast Care Center Navigator to an APN.

N/R: Not Reported.

Arnall et al. (2019) explored the benefits of including a pharmacist in the PM team, including continual updating and maintenance of the patient’s medication list, input into available therapies, and providing additional supportive care to the patient (Arnall et al., 2019). Similarly, Fu et al. (2020) identified the benefits of engaging nurses in pharmacogenetics and pharmacogenomics and concluded that nurses could use PM to effectively provide care to the patient. The study recommended funding to build the capacity of nurses to conduct PM research and integration of PM concepts and skills into nursing education (Fu et al., 2020). Bol and Meric-Bernstam (2015) focused on the structure of PM and highlighted the benefits of a multi-disciplinary team (MDT) for consensual decision-making. MDTs would build on and combine the skills and expertise areas of pathologists, medical oncologists, data analysts and informaticians, with surgeons acting as a bridge between all these professionals (Bol & Meric-Bernstam, 2015).

McAllister and Schmitt (2015) explored the engagement of a navigator (oncology nurse) to assist with the genetic profiling and decision-making process. The introduction of the navigator led to a reduction in test ordering and reporting turnaround from 26.3 to 11 days and 38 to 20 days respectively. This successful innovation led to a proposal to redesign the Registered Nurse Breast Care Centre Navigator (McAllister & Schmitt, 2015).

Borden et al. (2019) reviewed the use of an interactive clinical decision support (CDS) system that enabled the team to review up to date patient information and results and to make any medication changes influenced by the information presented. The medication changes were influenced by pharmacogenomics information in 42% of cases, and providers usually felt confident in the information provided by CDS (Borden et al., 2019).

Bokkers et al. (2022) focused on the implementation and benefits of an online training module that was designed specifically to educate HCPs. Their results demonstrated significant improvement in knowledge after 6 months of online training (*p* = 0.058) (Bokkers et al., 2022). Calzone et al. (2018) also explored the education of HCPs, with a focus on the pairing of a hospital administrator with an educator opinion leader. There was a statistically significant increment in awareness and intention to learn in the intervention group compared to the control group (*p* = 0.001) (Calzone et al., 2018).

Caraballo et al. (2017) focused on an intervention that consisted of eight interrelated functional components, which included institutional leadership support, education, governance, laboratory results, knowledge, implementation and long-term maintenance strategy. Furthermore, 11 educational resources on drug-gene interactions and five modules were developed and implemented after the success of the model. These resources were appreciated and welcomed by pharmacists and clinicians (Caraballo et al., 2017).

## Discussion

This review highlighted the current engagement of HCPs in PM and presented mixed findings on their knowledge, attitudes, and confidence levels. This variation could be due to the variability in the tools used to measure these factors, indicating an urgent need for future research to develop validated measurement tools. Notably, our scoping review finds a strong desire among the HCPs to further their education and training in PM. Addressing this need would improve the skills and confidence levels of HCPs around PM and address the lack of trained personnel (Al Bakir et al., 2019; Cusack et al., 2021; Diamonstein et al., 2018; Harding et al., 2019; Li et al., 2015; McCauley et al., 2017; Teng & Spigelman, 2014; Thavaneswaran et al., 2021).

The review also identified different structural barriers in the provision of PM. The most commonly reported barriers were the high cost of PM coupled with limited insurance coverage, lack of trained personnel to deliver PM services, lack of access to testing technology and facilities, and lack of clinical guidelines for providing PM. HCPs also shared concerns about discrimination of patients by insurers based on their genetic test results (Eum et al., 2018; Hann et al., 2017). There was geographic variation in the presence of these barriers. All of these barriers were reported in LMICs by Adeniji et al. (2021) and Pokharel et al. (2016). Table 5 provides further indications on geographical differences, although most studies captured in this scoping review were conducted in high-income countries (HICs), primarily in North America. While this may be an artefact of the location of the researchers studying PM, it more likely implies an unequal distribution in the use of PM globally. Most cancer research is conducted in HICs, despite evidence of differences at the molecular level of cancer in HICs and LMICs (Drake et al., 2018).

Additionally, the literature highlights that some LMICs lack skilled workforce to work with PM, both due to the lack of training and ‘braindrain’, that is, skilled HCPs from LMICs leaving to work in HICs, further widening the global health inequities (Adeniji et al., 2021). Major improvements in health infrastructure and resources, such as training, are required for the direct transfer of PM models in LMICs as implemented in HICs (Drake et al., 2018). Personnel rotation and other knowledge sharing initiatives with regards to PM may also help to reduce the health gap between HICs and LMICs.

Studies also highlighted important differences between skills and knowledge of younger and older physicians (Hamilton et al., 2017; McCauley et al., 2017). As the use of PM is very likely to intensify in oncology and broaden out to further disease areas, it will be essential to upskill all HCPs through dedicated continuous assessment programmes and curriculum redesign where necessary in order to level out differences in confidence levels.

The economics of PM were indicated to be the major structural barrier. For instance, the therapy-only cost of Chimeric Antigen Receptor Therapies (CAR-T) ranges from US$373,000 to US$475,000 and is often not considered cost-effective for reimbursement in publicly funded health systems (Hamilton et al., 2017; Mitchell et al., 2019). Along with the cost of the therapy, there are added costs of transport, tests, and patient opportunity costs (Snyder et al., 2021). Hence, without a comprehensive restructuring of health insurance systems, PM will continue to be out of reach for numerous patients. A mechanism to tackle this could be through the introduction of PM produced in non-pharmaceutical settings, which has been shown to be clinically and economically effective, including in LMICs. However, there are legislative and marketing restrictions in the wider distribution of these therapies (Johnson, 2024). Further research on how to lower the considerable economic barriers to utilising PM is urgently required.

The innovative practices identified in this review focused on building capacity and diversity with the introduction of new personnel to the oncology team to cater to the patients’ needs. Additionally, the use of technology to assist with the decision-making process was also found to be effective. Their use showed that HCP teams have devised local means to address challenges posed by PM; they also give important pointers as to the broader applicability of such innovative practices. From a health systems perspective, such innovative practices must be assessed, and if deemed effective, rolled out more broadly. Gaps at the institutional and organisational level may be addressed if effective innovations are scaled up to ensure smoother provision of PM therapies to the public.

Compared with previous reviews, our study considered knowledge levels, barriers, and innovative practices as related variables, with the latter particularly important for future PM strategies and resource allocation (Delikurt et al., 2015; Walters et al., 2023). A further strength of this review is the inclusion of the range of alternative terms used to commonly define PM, which likely captured a wider set of studies. The study does have some limitations, one of them being the exclusion of grey literature. Further, most studies were from HICs, which could have influenced some of the results. The study captures articles from an almost ten-year timespan, but given the low numbers of studies involved we could not ascertain whether there was a marked shift in HCPs expertise and skills between the earlier and later studies. Given the rapid increase in research around PM, future research should seek to identify such trends where possible.

## Conclusion

PM is in increasing demand and offers clear benefits to medically eligible patients who can access it. However, health systems and organisations and HCPs are not fully ready to implement it with the existing infrastructures and skillsets. This is the case even in HICs, with gaps in skill level and health systems readiness exacerbated in LMICs. Urgent investment in technologies, infrastructure, and medical education is needed to support the provision of PM and upskilling of the current workforce to meet the demands for these therapies. Global structured knowledge sharing and peer learning practices may increase HCPs’ confidence levels, and a systematic mapping of innovative practices to overcome barriers would assist in increasing health systems readiness to fully embrace PM. Further research should assess the inter-connectedness of different barriers and also address legislative, financial and regulatory barriers to the introduction of PMs.

## Supplementary Material

Supplementary material _Search Strategy.docx

## Data Availability

All the required data have been made available through the tables and supplementary file.

## References

[CIT0001] Adejumo, P. O., Kolawole, I. O., Ojo, I. O., Ilesanmi, R. E., Olorunfemi, O., & Tijani, W. A. (2021). University students’ knowledge and readiness to practice genomic nursing in Nigeria. *International Journal of Africa Nursing Sciences*, *15*, 100371. 10.1016/j.ijans.2021.100371

[CIT0002] Ademuyiwa, F. O., Salyer, P., Tao, Y., Luo, J., Hensing, W. L., Afolalu, A., Peterson, L. L., Weilbaecher, K., Housten, A. J., Baumann, A. A., Desai, M., Jones, S., Linnenbringer, E., Plichta, J., & Bierut, L. (2021). Genetic counseling and testing in african american patients with breast cancer: A nationwide survey of US breast oncologists. *Journal of Clinical Oncology*, *39*(36), 4020–4028. 10.1200/jco.21.01426 34662201 PMC13524037

[CIT0003] Adeniji, A. A., Dulal, S., & Martin, M. G. (2021). Personalized medicine in oncology in the developing world: Barriers and concepts to improve status quo. *World Journal of Oncology*, *12*(2–3), 50–60. 10.14740/wjon1345 34046099 PMC8139741

[CIT0004] Al Bakir, I., Sebepos-Rogers, G. M., Burton, H., & Monahan, K. J. (2019). Mainstreaming of genomic medicine in gastroenterology, present and future: A nationwide survey of UK gastroenterology trainees. *BMJ Open*, *9*(10), e030505. 10.1136/bmjopen-2019-030505 PMC6830597 31640999

[CIT0005] Albitar, L., & Abou Alchamat, G. (2021). Pharmacogenetics: Knowledge assessment amongst Syrian pharmacists and physicians. *BMC Health Services Research*, *21*(1), 1031. 10.1186/s12913-021-07040-9 34592972 PMC8485485

[CIT0006] Ali-Khan, S., Kowal, S., & Luth, W. (2016). *Terminology for personalized medicine: A systematic collection*. PACEOMICS.

[CIT0007] American Cancer Society. (2022). What is precision medicine. American Cancer Society. Retrieved August 22 from https://www.cancer.org/treatment/treatments-and-side-effects/treatment-types/precision-medicine.html

[CIT0008] Anderson, E. C., Hinton, A. C., Lary, C. W., Fenton, A., Antov, A., Edelman, E., Helbig, P., Reed, K., Miesfeldt, S., Thomas, C. A., Hall, M. J., Roberts, J. S., Rueter, J., & Han, P. K. J (2021). Community oncologists’ perceptions and utilization of large-panel genomic tumor testing. *BMC Cancer*, *21*(1), 1273. 10.1186/s12885-021-08985-0 34823486 PMC8620967

[CIT0009] Aomori, T., Sakurai, H., & Nishihara, H. (2022). Cancer genomic medicine in Japan and the roles of pharmacists. *Pharmacogenetics and Genomics*, *32*(6), 242–245. 10.1097/fpc.0000000000000476 35696282

[CIT0010] Arksey, H., & O’Malley, L. (2005). Scoping studies: Towards a methodological framework. *International Journal of Social Research Methodology*, *8*(1), 19–32. 10.1080/1364557032000119616

[CIT0011] Arnall, J. R., Petro, R., Patel, J. N., & Kennedy, L. (2019). A clinical pharmacy pilot within a Precision Medicine Program for cancer patients and review of related pharmacist clinical practice. *Journal of Oncology Pharmacy Practice*, *25*(1), 179–186. 10.1177/1078155217738324 29078708

[CIT0012] Ashley, E. A. (2015). The precision medicine initiative: A new national effort. *JAMA*, *313*(21), 2119–2120. 10.1001/jama.2015.3595 25928209

[CIT0013] Bazarbashi, S., Alsharm, A., Meshref, A., Mrabti, H., Ansari, J., Ghosn, M., Abdulla, M., & Urun, Y. (2022). Management of metastatic castration-resistant prostate cancer in Middle East African countries: Challenges and strategic recommendations. *Urology Annals*, *14*(4), 303–313. 10.4103/ua.ua_148_21 36505997 PMC9731188

[CIT0014] Bokkers, K., Zweemer, R. P., Koudijs, M. J., Stehouwer, S., Velthuizen, M. E., Bleiker, E. M. A., & Ausems, M. (2022). Positive experiences of healthcare professionals with a mainstreaming approach of germline genetic testing for women with ovarian cancer. *Familial Cancer*, *21*(3), 295–304. 10.1007/s10689-021-00277-7 34617209 PMC9203381

[CIT0015] Bol, G. M., & Meric-Bernstam, F. (2015). The role of surgeons in building a personalized medicine program. *Journal of Surgical Oncology*, *111*(1), 3–8. 10.1002/jso.23684 24964977 PMC4285684

[CIT0016] Bombard, Y., Rozmovits, L., Trudeau, M., Leighl, N. B., Deal, K., & Marshall, D. A. (2015). The value of personalizing medicine: Medical oncologists’ views on gene expression profiling in breast cancer treatment. *The Oncologist*, *20*(4), 351–356. 10.1634/theoncologist.2014-0268 25746345 PMC4391763

[CIT0017] Borden, B. A., Galecki, P., Wellmann, R., Danahey, K., Lee, S. M., Patrick-Miller, L., Sorrentino, M. J., Nanda, R., Koyner, J. L., Polonsky, T. S., Stadler, W. M., Mulcahy, C., Kavitt, R. T., Ratain, M. J., Meltzer, D. O., & O’Donnell, P. H. (2019). Assessment of provider-perceived barriers to clinical use of pharmacogenomics during participation in an institutional implementation study. *Pharmacogenetics and Genomics*, *29*(2), 31–38. 10.1097/FPC.0000000000000362 30531377

[CIT0018] Braun, V., & Clarke, V. (2006). Using thematic analysis in psychology. *Qualitative Research in Psychology*, *3*(2), 77–101. 10.1191/1478088706qp063oa

[CIT0019] Brittain, H. K., Scott, R., & Thomas, E. (2017). The rise of the genome and personalised medicine. *Clinical Medicine*, *17*(6), 545–551. 10.7861/clinmedicine.17-6-545 29196356 PMC6297695

[CIT0020] Calzone, K. A., Jenkins, J., Culp, S., & Badzek, L. (2018). Hospital nursing leadership-led interventions increased genomic awareness and educational intent in Magnet settings. *Nursing Outlook*, *66*(3), 244–253. 10.1016/j.outlook.2017.10.010 29544651 PMC5949252

[CIT0021] Caraballo, P. J., Hodge, L. S., Bielinski, S. J., Stewart, A. K., Farrugia, G., Schultz, C. G., Rohrer-Vitek, C. R., Olson, J. E., St Sauver, J. L., Roger, V. L., Parkulo, M. A., Kullo, I. J., Nicholson, W. T., Elliott, M. A., Black, J. L., & Weinshilboum, R. M. (2017). Multidisciplinary model to implement pharmacogenomics at the point of care. *Genetics in Medicine*, *19*(4), 421–429. 10.1038/gim.2016.120 27657685 PMC5362352

[CIT0022] Carroll, J. C., Allanson, J., Morrison, S., Miller, F. A., Wilson, B. J., Permaul, J. A., & Telner, D. (2019). Informing integration of genomic medicine into primary care: An assessment of current practice, attitudes, and desired resources. *Frontiers in Genetics*, *10*, 1189. 10.3389/fgene.2019.01189 31824576 PMC6882282

[CIT0023] Cho, H. N., Shin, S. Y., Hwangbo, B., Chang, Y. J., Cho, J., Kong, S. Y., Choi, K. S., & Lee, E. S. (2020). Views on precision medicine among health professionals in Korea: A mixed methods study. *Yonsei Medical Journal*, *61*(2), 192–197. 10.3349/ymj.2020.61.2.192 31997629 PMC6992457

[CIT0024] Ciardiello, F., Adams, R., Tabernero, J., Seufferlein, T., Taieb, J., Moiseyenko, V., Ma, B., Lopez, G., Vansteenkiste, J. F., Esser, R., & Tejpar, S. (2016). Awareness, understanding, and adoption of precision medicine to deliver personalized treatment for patients with cancer: A multinational survey comparison of physicians and patients. *The Oncologist*, *21*(3), 292–300. 10.1634/theoncologist.2015-0279 26888693 PMC4786350

[CIT0025] Cusack, M. B., Hickerton, C., Nisselle, A., McClaren, B., Terrill, B., Gaff, C., Dunlop, K., & Metcalfe, S. (2021). General practitioners’ views on genomics, practice and education A qualitative interview study. *Australian Journal of General Practice*, *50*(10), 747–752. 10.31128/AJGP-05-20-5448 34590089

[CIT0026] De Abreu Lourenco, R., McCarthy, M. C., McMillan, L. J., Sullivan, M., & Gillam, L. (2021). Understanding decisions to participate in genomic medicine in children’s cancer care: A comparison of what influences parents, health care providers, and the general community. *Pediatric Blood & Cancer*, *68*(8), e29101. 10.1002/pbc.29101 34089211

[CIT0027] De Grandis, G., & Halgunset, V. (2016). Conceptual and terminological confusion around personalised medicine: A coping strategy. *BMC Medical Ethics*, *17*(1), 43. 10.1186/s12910-016-0122-4 27431285 PMC4950113

[CIT0028] Delikurt, T., Williamson, G. R., Anastasiadou, V., & Skirton, H. (2015). A systematic review of factors that act as barriers to patient referral to genetic services. *European Journal of Human Genetics*, *23*(6), 739–745. 10.1038/ejhg.2014.180 25205405 PMC4795051

[CIT0029] Diamonstein, C., Stevens, B., Hashmi, S. S., Refuerzo, J., Sullivan, C., & Hoskovec, J. (2018). Physicians’ awareness and utilization of genetic services in Texas. *Journal of Genetic Counseling*, *27*(4), 968–977. 10.1007/s10897-017-0199-z 29280038

[CIT0030] Dias, M. M., Ward, H. M., Sorich, M. J., & McKinnon, R. A. (2014). Exploration of the perceptions, barriers and drivers of pharmacogenomics practice among hospital pharmacists in Adelaide, South Australia. *The Pharmacogenomics Journal*, *14*(3), 235–240. 10.1038/tpj.2013.31 24018620

[CIT0031] Drake, T. M., Knight, S. R., Harrison, E. M., & Søreide, K. (2018). Global inequities in precision medicine and molecular cancer research. *Frontiers in Oncology*, *8*, 346. 10.3389/fonc.2018.00346 30234014 PMC6131579

[CIT0032] Eum, H., Lee, M., Yoon, J., Cho, J., Lee, E. S., Choi, K. S., Lee, S., Jung, S. Y., Lim, M. C., Kong, S. Y., & Chang, Y. J. (2018). Differences in attitudes toward genetic testing among the public, patients, and health-care professionals in Korea. *European Journal of Human Genetics*, *26*(10), 1432–1440. 10.1038/s41431-018-0191-6 29915183 PMC6138694

[CIT0033] Farmaki, A., Manolopoulos, E., & Natsiavas, P. (2024). Will precision medicine meet digital health? A systematic review of pharmacogenomics clinical decision support systems used in clinical practice. *Omics: A Journal of Integrative Biology*, *28*(9), 442–460. 10.1089/omi.2024.0131 39136110

[CIT0034] Fountzilas, E., Apostolou, P., Vasiliadis, A. V., Aivazi, D., Saloustros, E., & Fostira, F. (2022). Physicians’ experience, practice and education, on genetic testing and genetic counseling: A nationwide survey study in Greece. *Familial Cancer*, *21*(4), 479–487. 10.1007/s10689-022-00290-4 35067824

[CIT0035] Fu, M. R., Kurnat-Thoma, E., Starkweather, A., Henderson, W. A., Cashion, A. K., Williams, J. K., Katapodi, M. C., Reuter-Rice, K., Hickey, K. T., Barcelona de Mendoza, V., Calzone, K., Conley, Y. P., Anderson, C. M., Lyon, D. E., Weaver, M. T., Shiao, P. K., Constantino, R. E., Wung, S. F., Hammer, M. J., Voss, J. G., & Coleman, B. (2020). Precision health: A nursing perspective. *International Journal of Nursing Sciences*, *7*(1), 5–12. 10.1016/j.ijnss.2019.12.008 32099853 PMC7031154

[CIT0036] Gameiro, G. R., Sinkunas, V., Liguori, G. R., & Auler-Júnior, J. O. C. (2018). Precision medicine: Changing the way we think about healthcare. *Clinics*, *73*, e723. 10.6061/clinics/2017/e723 30517307 PMC6251254

[CIT0037] Hall, M. J., Forman, A. D., Montgomery, S. V., Rainey, K. L., & Daly, M. B. (2015). Understanding patient and provider perceptions and expectations of genomic medicine. *Journal of Surgical Oncology*, *111*(1), 9–17. 10.1002/jso.23712 24992205 PMC4286413

[CIT0038] Hamilton, J., Abdiwahab, E., Edwards, H., Fang, M.-L., Jdayani, A., Breslau, E., Hamilton, J. G., Edwards, H. M., & Breslau, E. S. (2017). Primary care providers’ cancer genetic testing-related knowledge, attitudes, and communication behaviors: A systematic review and research agenda. *Journal of General Internal Medicine*, *32*(3), 315–324. 10.1007/s11606-016-3943-4 27995427 PMC5331015

[CIT0039] Hamilton, J. G., Banerjee, S. C., Carlsson, S. V., Vera, J., Lynch, K. A., Sar-Graycar, L., Martin, C. M., Parker, P. A., & Hay, J. L. (2021). Clinician perspectives on communication and implementation challenges in precision oncology. *Personalized Medicine*, *18*(6), 559–572. 10.2217/pme-2021-0048 34674550 PMC8607478

[CIT0040] Hann, K. E. J., Fraser, L., Side, L., Gessler, S., Waller, J., Erson, S. C., Freeman, M., Jacobs, I., & Lanceley, A. (2017). Health care professionals’ attitudes towards population-based genetic testing and risk-stratification for ovarian cancer: A cross-sectional survey. *BMC Women’s Health*, *17*(1), 132. 10.1186/s12905-017-0488-6 29246147 PMC5732525

[CIT0041] Harding, B., Webber, C., Ruhl, L., Dalgarno, N., Armour, C. M., Birtwhistle, R., Brown, G., Carroll, J. C., Flavin, M., Phillips, S., & MacKenzie, J. J. (2019). Primary care providers’ lived experiences of genetics in practice. *Journal of Community Genetics*, *10*(1), 85–93. 10.1007/s12687-018-0364-6 29700759 PMC6325046

[CIT0042] Hinderer, M., Boeker, M., Wagner, S. A., Binder, H., Ückert, F., Newe, S., Hülsemann, J. L., Neumaier, M., Schade-Brittinger, C., Acker, T., Prokosch, H. U., & Sedlmayr, B. (2017). The experience of physicians in pharmacogenomic clinical decision support within eight German university hospitals. *Pharmacogenomics*, *18*(8), 773–785. 10.2217/pgs-2017-0027 28593816

[CIT0043] Jackson, S. E., & Chester, J. D. (2015). Personalised cancer medicine. *International Journal of Cancer*, *137*(2), 262–266. 10.1002/ijc.28940 24789362

[CIT0044] Johnson, B. (2024). Reducing the costs of blockbuster gene and cell therapies in the Global South. *Nature Biotechnology*, *42*(1), 8–12. 10.1038/s41587-023-02049-3 38212491

[CIT0045] Kolesar, J. M., & Vermeulen, L. C. (2021). Precision medicine: Opportunities for health-system pharmacists. *American Journal of Health-System Pharmacy*, *78*(11), 999–1003. 10.1093/ajhp/zxab084 33693532 PMC7989629

[CIT0046] Komatsu, H., & Yagasaki, K. (2014). Are we ready for personalized cancer risk management? The view from breast-care providers. *International Journal of Nursing Practice*, *20*(1), 39–45. 10.1111/ijn.12115 24580974

[CIT0047] Levac, D., Colquhoun, H., & O’Brien, K. K. (2010). Scoping studies: Advancing the methodology. *Implementation Science*, *5*(1), 69. 10.1186/1748-5908-5-69 20854677 PMC2954944

[CIT0048] Li, J., Xu, T. D., & Yashar, B. M. (2015). Genetics educational needs in China: Physicians’ experience and knowledge of genetic testing. *Genetics in Medicine*, *17*(9), 757–760. 10.1038/gim.2014.182 25503494

[CIT0049] McAllister, K. A., & Schmitt, M. L. (2015). Impact of a nurse navigator on genomic testing and timely treatment decision making in patients with breast cancer. *Clinical Journal of Oncology Nursing*, *19*(5), 510–512. 10.1188/15.CJON.510-512 26414569

[CIT0050] McCauley, M. P., Marcus, R. K., Strong, K. A., Visotcky, A. M., Shimoyama, M. E., & Derse, A. R. (2017). Genetics and genomics in clinical practice: The views of Wisconsin physicians. *WMJ*, *116*(2), 69–74. 29323820

[CIT0051] Mishra, V., Chanda, P., Tambuwala, M. M., & Suttee, A. (2019). Personalized medicine: An overview. *International Journal of Pharmaceutical Quality Assurance*, *10*(02), 290–294. 10.25258/ijpqa.10.2.13

[CIT0052] Mitchell, D., Kenderian, S., Rajkumar, S. V. (2019). Letting academic medical centers make CAR-T drugs would save billions. STAT. Retrieved March 24 from https://www.statnews.com/2019/11/20/car-t-drugs-academic-medical-centers-save-billions/

[CIT0053] Nagy, M., Lynch, M., Kamal, S., Mohamed, S., Hadad, A., Abouelnaga, S., & Aquilante, C. L. (2020). Assessment of healthcare professionals’ knowledge, attitudes, and perceived challenges of clinical pharmacogenetic testing in Egypt. *Personalized Medicine*, *17*(4), 251–260. 10.2217/pme-2019-0163 32589096

[CIT0054] Nagy, M., Tsermpini, E. E., Siamoglou, S., & Patrinos, G. P. (2020). Evaluating the current level of pharmacists’ pharmacogenomics knowledge and its impact on pharmacogenomics implementation. *Pharmacogenomics*, *21*(16), 1179–1189. 10.2217/pgs-2020-0076 33118449

[CIT0055] Ong, C. S. B., Fok, R. W., Tan, R. C. A., Fung, S. M., Sun, S., & Ngeow, J. Y. Y. (2022). General practitioners’ (GPs) experience, attitudes and needs on clinical genetic services: A systematic review. *Family Medicine and Community Health*, *10*(4), e001515. 10.1136/fmch-2021-001515 PMC9717000 36450397

[CIT0056] Peters, M., Godfrey, C., McInerney, P., Soares, C., Khalil, H., & Parker, D. (2015). The *Joanna Briggs Institute reviewers’ manual 2015: Methodology for JBI scoping reviews*. https://reben.com.br/revista/wp-content/uploads/2020/10/Scoping.pdf

[CIT0057] Pokharel, H. P., Hacker, N. F., & Andrews, L. (2016). Genetic testing in a gynaecological oncology care in developing countries-knowledge, attitudes and perception of Nepalese clinicians. *Gynecologic Oncology Research and Practice*, *3*, 12. 10.1186/s40661-016-0034-5 27980798 PMC5137212

[CIT0058] Przybylski, D. J., Dow-Hillgartner, E. N., Reed, M. P., & Fallon, M. J. (2020). Current state assessment survey of challenges of pharmacogenomics within oncology pharmacy practice. *Journal of Oncology Pharmacy Practice*, *26*(6), 1374–1381. 10.1177/1078155219896395 31937189

[CIT0059] Raedler, L. A. (2015). Keytruda (pembrolizumab): First PD-1 inhibitor approved for previously treated unresectable or metastatic melanoma. *American Health & Drug Benefits*, *8*(Spec Feature), 96–100. 26629272 PMC4665064

[CIT9967356] Roberts, M. C., Bryson, Amy., Weinberger, M., Dusetzina, S. B., Dinan, M. A., Reeder-Hayes, K., & Wheeler, S. B. (2016). Oncologists’ barriers and facilitators for oncotype dx use: Qualitative study. *International Journal of Technology Assessment in Health Care*, *32*(5), 355–361. 10.1017/S026646231600060X 27958190 PMC6526532

[CIT0060] Shelton, C. A., & Whitcomb, D. C. (2015). Evolving roles for physicians and genetic counselors in managing complex genetic disorders. *Clinical and Translational Gastroenterology*, *6*(11), e124. 10.1038/ctg.2015.46 26561988 PMC4817528

[CIT0061] Smit, A. K., Sharman, A. R., Espinoza, D., Wallingford, C., Young, M. A., Dunlop, K., Tiller, J., Newson, A. J., Meiser, B., Cust, A. E., & Yanes, T. (2021). Knowledge, views and expectations for cancer polygenic risk testing in clinical practice: A cross-sectional survey of health professionals. *Clinical Genetics*, *100*(4), 430–439. 10.1111/cge.14025 34216141

[CIT0062] Snyder, S., Albertson, T., Garcia, J., Gitlin, M., & Jun, M. P. (2021). Travel-related economic burden of chimeric antigen receptor T cell therapy administration by site of care. *Advances in Therapy*, *38*(8), 4541–4555. 10.1007/s12325-021-01839-y 34279805 PMC8342383

[CIT0063] Spanakis, M., Patelarou, A. E., & Patelarou, E. (2020). Nursing personnel in the era of personalized healthcare in clinical practice. *Journal of Personalized Medicine*, *10*(3), 56. 10.3390/jpm10030056 32610469 PMC7565499

[CIT0064] Teng, I., & Spigelman, A. (2014). Attitudes and knowledge of medical practitioners to hereditary cancer clinics and cancer genetic testing. *Familial Cancer*, *13*(2), 311–324. 10.1007/s10689-013-9695-y 24306515

[CIT0065] Thavaneswaran, S., Ballinger, M., Butow, P., Meiser, B., Goldstein, D., Lin, F., Napier, C., Thomas, D., & Best, M. (2021). The experiences and needs of Australian medical oncologists in integrating comprehensive genomic profiling into clinical care: A nation-wide survey. *Oncotarget*, *12*(21), 2169–2176. 10.18632/oncotarget.28076 34676049 PMC8522847

[CIT0066] Tricco, A. C., Lillie, E., Zarin, W., O’Brien, K. K., Colquhoun, H., Levac, D., Moher, D., Peters, M. D. J., Horsley, T., Weeks, L., Hempel, S., Akl, E. A., Chang, C., McGowan, J., Stewart, L., Hartling, L., Aldcroft, A., Wilson, M. G., Garritty, C., … Straus, S. E. (2018). PRISMA extension for scoping reviews (PRISMA-ScR): Checklist and explanation. *Annals of Internal Medicine*, *169*(7), 467–473. 10.7326/M18-0850 30178033

[CIT0067] Vashistha, V., Poonnen, P. J., Snowdon, J. L., Skinner, H. G., McCaffrey, V., Spector, N. L., Hintze, B., Duffy, J. E., Weeraratne, D., Jackson, G. P., Kelley, M. J., & Patel, V. L. (2020). Medical oncologists’ perspectives of the Veterans Affairs National Precision Oncology Program. *PloS One*, *15*(7), e0235861. 10.1371/journal.pone.0235861 32706774 PMC7380614

[CIT0068] Vetsch, J., Wakefield, C. E., Techakesari, P., Warby, M., Ziegler, D. S., O’Brien, T. A., Drinkwater, C., Neeman, N., & Tucker, K. (2019). Healthcare professionals’ attitudes toward cancer precision medicine: A systematic review. *Seminars in Oncology*, *46*(3), 291–303. 10.1053/j.seminoncol.2019.05.001 31221444

[CIT0069] Walters, S., Aldous, C., & Malherbe, H. (2023). Healthcare practitioners’ knowledge, attitudes and practices of genetics and genetic testing in low-or middle-income countries-A scoping review. *Journal of Community Genetics*, *15*(5), 461–474. 10.1007/s12687-024-00721-y PMC11549072 39120782

[CIT190004] Wevers, M. R., Aaronson, N. K., Bleiker, E. M. A., Hahn, D. E. E., Brouwer, T., Van Dalen, T., Theunissen, E. B., Van Ooijen, B., De Roos, M. A., Borgstein, P. J., Vrouenraets, B. C., Vriens, E., Bouma, W. H., Rijna, H., Vente, J. P., Kuenen, M. A., Van Der Sanden-Melis, J., Witkamp, A. J., Rutgers, E. J. T., Verhoef, S., & Ausems, M. G. E. M. (2017). Rapid genetic counseling and testing in newly diagnosed breast cancer: Patients’ and health professionals’ attitudes, experiences, and evaluation of effects on treatment decision making. *Journal of Surgical Oncology*, *116*(8), 1029–1039. 10.1002/jso.24763 28703900

