# Supplementary material for: Exploring healthcare personnel’s knowledge, barriers, and innovative approaches in personalised oncology medicine: a scoping review
Source: Crit Public Health. 2025 Jun 28;35(1):2520410. doi: 10.1080/09581596.2025.2520410 (PMC12315847; doi:10.1080/09581596.2025.2520410)
Supplement: Supplementary material _Search Strategy.docx [file CCPH_A_2520410_SM7046.docx]

Sample search strategy for PubMed

**(("Health Personnel"[Mesh:NoExp] OR "Health Personnel*" OR "health care personnel*" OR "healthcare personnel" OR "health care provider*" OR "healthcare provider*" OR "health provider*" OR "healthcare worker*" OR "Health worker*" OR "health care worker*" OR "health care professional*" OR "healthcare professional*" OR "health profession personnel*" OR "health care practitioner*" OR "healthcare practitioner*" OR "health practitioner*" OR "medical personnel*" OR "Physicians, Primary Care"[Mesh:NoExp] OR "Nurses"[Mesh:NoExp] OR "nurse*" OR "doctor*" OR "Physicians"[Mesh:NoExp] OR "physician*" OR "Physicians, Family"[Mesh:NoExp] OR "Pharmacists"[Mesh:NoExp] OR "pharmacist*" OR "Oncologists"[Mesh:NoExp] OR "oncologist*" OR "pathologist*" OR "General Practitioners"[Mesh:NoExp] OR "general practitioner*" OR "GP" OR "family physician*") AND ("Precision Medicine"[Mesh:NoExp] OR "Genomic Medicine"[Mesh:NoExp] OR "genetic medicine*" OR "Pharmacogenomic*"OR "Pharmacogenetics"[Mesh:NoExp] OR "Pharmacogenetic*" OR "Personalised medicine*" OR "personalized medicine" OR "Individualised medicine*" OR "individualized medicine*" OR "genetic screening" OR "Genetic Testing"[Mesh:NoExp] OR "genetic testing*" OR "Predictive medicine*" OR "precision cancer medicine*" OR "precision therap*" OR "individualised therap*" OR "individualized therap*" OR "personalised therap*" OR "personalized therap*" OR "cell and gene therap*" OR "Genetic Therapy"[Mesh:NoExp] OR "gene therap*" OR "stratified medicine*")) AND ("Neoplasms"[Mesh] OR "tumor*" OR "neoplasm*" OR "tumour*" OR "neoplasia*" OR "cancer*" OR "malignanc*" OR "malignant neoplastic disease")** Filters: **English, from 2014 – 2023**
